# Improving the Duration and Rate of Home-Based Kangaroo Mother Care: A Before-and-After Intervention Study

**DOI:** 10.7759/cureus.37861

**Published:** 2023-04-20

**Authors:** Meena Patawat, Ramesh Choudhary, Mahendra K Jain, Roshan Chanchalani, Anubhuti Jain

**Affiliations:** 1 Department of Pediatrics, Government Medical College, Pali, IND; 2 Department of Paediatrics, JK Lon Hospital, Sawai Man Singh (SMS) Medical College, Jaipur, IND; 3 Department of Neonatology, All India Institutes of Medical Sciences (AIIMS) Bhopal, Bhopal, IND; 4 Department of Pediatrics Surgery, All India Institutes of Medical Sciences (AIIMS) Bhopal, Bhopal, IND; 5 Department of Obstetrics and Gynaecology, Jain Clinic, Bhopal, IND

**Keywords:** needs analysis, home-based kangaroo mother care, low birth weight, prematurity, neonatal hypothermia

## Abstract

Introduction

Kangaroo mother care (KMC) is an evidence-based, simple, time-tested, low-cost, and high-impact intervention for neonatal survival in hospitals and the community, particularly in resource-constrained areas. This has many beneficial effects on sick and stable low-birth-weight babies, lactating mothers, families, society, and the government. However, despite the World Health Organization (WHO) and United Nations International Children's Emergency Fund (UNICEF) recommendations for KMC, there is no satisfactory implementation of it in the community as well as in facilities. This study aimed to improve the duration of home-based kangaroo mother care (HBKMC).

Material and methods

We conducted a before-and-after intervention hospital-based, single-center study in a level III neonatal intensive care unit (NICU) to improve the duration of HBKMC. The KMC duration was classified into four categories: short, extended, long, and continuous where KMC was provided for 4 hours/day, 5-8 hours/day, 9-12 hours/day, and more than 12 hours/day, respectively. All neonates with birth weight < 2.0 kg and their mothers/alternate KMC providers at a tertiary care hospital in India in the time period of five months from April 2021 to July 2021 were considered eligible for the study. We tested three sets of interventions by using the plan-do-study-act cycle (PDSA cycle). The first set of interventions was the sensitization of parents and healthcare workers regarding the benefits of KMC by comprehensive counseling to mothers and other family members using educational lectures, videos, charts, and posters. The second set of interventions was to reduce maternal anxiety/stress while maintaining maternal privacy by providing more female staff and teaching proper gown-wearing techniques. The third set of interventions was to solve lactation and environment temperature issues by providing antenatal and postnatal lactation counseling and warming of the nursery. The paired T-test and one-way analysis of variance (ANOVA) were used for statistical analysis, and p<0.05 was taken as significant.

Results

One hundred and eighty neonates were enrolled along with their mothers/alternate KMC providers in four phases, and three PDSA cycles were implemented. Out of 180 LBW infants, 21 (11.67%) infants received KMC < 4 hours/day. According to the KMC classification, 31% have continuous KMC in the institution, followed by 24% long KMC, 26% extended KMC, and 18% short KMC. After three PDSA cycles, HBKMC was 38.88% continuous KMC, followed by 24.22% long KMC, 20.55% extended KMC, and 16.11% short KMC. Continuous KMC was improved from 21% to 46% at the institute and 16% to 50% at home from phase 1 to phase 4 of the study after the implementation of three sets of interventions in three PDSA cycles. The phase-by-phase KMC rate and duration were improved after the application of the PDSA cycles, and this was maintained in HBKMC as well, but it was statistically not significant.

Conclusion

Sets of intervention packages based on needs analysis using the PDSA cycle were able to improve the rate and duration of KMC in the hospital and at home.

## Introduction

According to a recent World Health Organization (WHO) fact sheet, approximately 2.4 million newborns die each year worldwide, and most neonatal deaths occur in low-middle-income countries. India has the highest number of neonatal deaths in the world i.e. around 0.5 million [1]. According to the 2017 United Nations International Children's Emergency Fund (UNICEF) report, globally neonatal mortality contributes to about 50% of under 5 child mortality, and southern Asia and sub-Saharan Africa contribute to about 80% of total neonatal deaths worldwide. In fact, India alone contributes 24% of the total neonatal mortality worldwide [2].

Kangaroo mother care (KMC) is an evidence-based, simple, time-tested, low-cost, and high-impact intervention that reduces mortality by about 40% in preterm and low-birth-weight infants (LBWIs) and reduces nosocomial and neonatal sepsis by about 65% in LBWIs. Moreover, if immediate kangaroo mother care (IKMC) is implemented, the 13% neonatal mortality rate can be further reduced [3]. It brings a successful breastfeeding rate, good weight gain, and a shorter duration of hospital stay. 

Evidence from subsequently updated reviews by these authors supports the use of KMC in LBWIs as an adjunct and alternative to conventional medical care, particularly in resource-limited settings. Conventional mode of care (CMC) is a more advanced level reserved for the sick and premature LBWIs. CMC provides modern and high-cost equipment such as incubators, cardiopulmonary monitors, oximetry, intravenous infusion, and equipment such as continuous positive airway pressure (CPAP) machines and ventilators, which require highly trained manpower [4-7]. KMC is a safe, cost-effective, successful intervention with outcomes comparable to CMC, especially in resource-limited settings. In addition, it enhances parental involvement [8,9]. KMC is strongly recommended by the WHO as one of the 10 recommendations to improve the outcome of premature birth. The WHO states that KMC should be provided to all newborns less than 2000 grams of birth weight as soon as LBWIs are clinically stable [10]. However, despite the many benefits, the rate and duration of KMC are very low. This before-and-after intervention study was conducted to improve the duration and rate of home-based KMC by implementing sets of interventions based on needs analysis.

## Materials and methods

This was a single-center, before-and-after intervention study conducted between April 2021 and July 2021 at the neonatal intensive care unit (NICU) attached to the Department of Pediatric Medicine, JK Lon Hospital, SMS Medical College, Jaipur. All neonates with birth weight less than 2.0 kilograms (kg) and their mothers/alternate KMC providers were considered eligible for the study. An alternate KMC provider is any family member who is ready to spend quality time for KMC other than the mother [11,12].

The study excludes newborns with fatal congenital anomalies, mothers with severe diseases causing disability, alternate KMC providers not available, and families not willing to participate. Informed written consent for the study was obtained from the parents for enrolment into the study after explaining the purpose of the study. They were also provided with a participant information sheet. The study was approved by the office of the ethics committee SMS Medical College and attached Hospital, Jaipur, India (Reference Number- 346/MC/EC/2021, Date - 24th March 2021).

**Figure 1:**
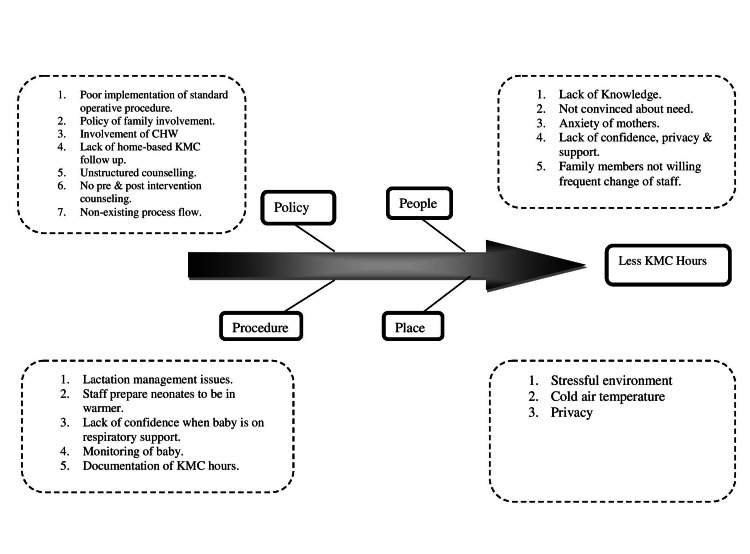
Fish Bone Analysis KMC: Kangaroo mother care; CHW: community health care worker

All mothers/alternative KMC providers whose newborns weighed less than 2 kg at birth were asked for enrollment in the study in NICU in JK Lon Hospital, SMS Medical College Jaipur. Counseling sessions were started at the pre-decided schedule post-admission while the baby was being stabilized in the hospital. Mother/alternative KMC provider-Baby dyads were classified as A, B, C, and D based on the last 48 hours of KMC performance. The KMC duration was classified into four categories: short, extended, long, and continuous where KMC provided 4 hours/day, 5-8 hours/day, 9-12 hours/day, and more than 12 hours/day respectively [13]. Before embarking on the study, we carried out a fishbone analysis, and a series of plan-do-study-act (PDSA) cycles were planned based on the needs analysis (Figure 1). We organized orientation sessions for nurses, doctors, and community health care workers (CHWs). A needs analysis was done on the basis of the detailed predesigned questionnaire regarding issues and constraints in the execution of KMC in the Institute and home-based kangaroo mother care (HBKMC).

The study was planned in four phases. Phase 1 was implemented on the basis of already existing policies and procedures regarding KMC in our institute. After phase 1, the first cycle of PDSA was applied during their hospital stay in the first and second weeks. Comprehensive counseling of the mother and other family members was done using educational lectures, videos, charts, and posters, and other family members were also allowed to participate in the KMC. Here, KMC was mentioned as part of the medical treatment. Then on the basis of needs analysis, the next two PDSA cycles and three more phases were carried out (Table 1). Based on PDSA cycles 1 and 2, two major issues were found i.e. lack of family support and household chores in the community for HBKMC. Based on the above community, healthcare workers were enrolled in the third PDSA to assist and counsel the mother and other family members. WhatsApp groups were also created, and online sessions were started to keep in constant touch with the CHWs and KMC providers. The infants and their parents were called for a detailed examination of the infants and the parent interview at three months of age. Baseline parental and neonatal data were recorded in the Performa sheet. All the data were uploaded into the Excel Sheet (Microsoft Excel 2008), and analysis was performed using IBM SPSS Statistics for Windows, Version 12 (Released 2004; IBM Corp., Armonk, New York, United States). Continuous data would be summarized in the form of mean and standard deviation. Continuous data would be expressed in the form of proportions. Differences in the proportion would be analyzed using the Chi-Square test. The difference in mean was analyzed using an unpaired t-test. Comparison of numerical data across four study groups was carried out by using one-way analysis of variance (ANOVA) (parametric ANOVA or non-parametric Kruskal-Wallis ANOVA) depending on the distribution of data. The paired t-test is used to test whether the mean difference between pairs of measurements is zero or not. The level of significance would be kept at 95% for all statistical analyses. P value <0.05 was taken as significant.

**Table 1:** Phases and Plan-Do-Study-Act Cycle KMC: Kangaroo mother care; CHW: community health care worker; PDSA: plan-do-study-act

PDSA Cycle	Time line	Plan	Do	Study	Act
PDSA 1	1^st^ and 2^nd^ week (16 April 2021 to 30 April 2021)	Sensitization of health care workers and parents regarding benefits of KMC.	Comprehensive counseling to mother and other family members using educational lectures, videos, charts, and posters. Residents should mention KMC as a part of daily treatment. Other family members to be allowed to take part in KMC	Average KMC duration increased to more than four hours per day	Adopt the PDSA cycle and continue with it
PDSA 2	3^rd^ and 4^th^ weeks (01 May 2021 to 15 May 2021)	To reduce mother’s stress and anxiety and to maintain their privacy	More female staff to be appointed in KMC block proper gown wearing technique to be taught	Average KMC duration Increased to more than six hours per day	Adopt the PDSA cycle, ongoing felicitation and continue with it.
PDSA 3	5^th^ week onwards (16 May 2021- 15 August 2021)	To resolve lactation issues and environment temperature issues	Enrolling CHWs to help at community level lactational counselor, warming of nursery, and pre and post-natal counseling	KMC duration remained sustained at > 6 h	Adopt the PDSA cycle and sustain with it.

## Results

**Figure 2:**
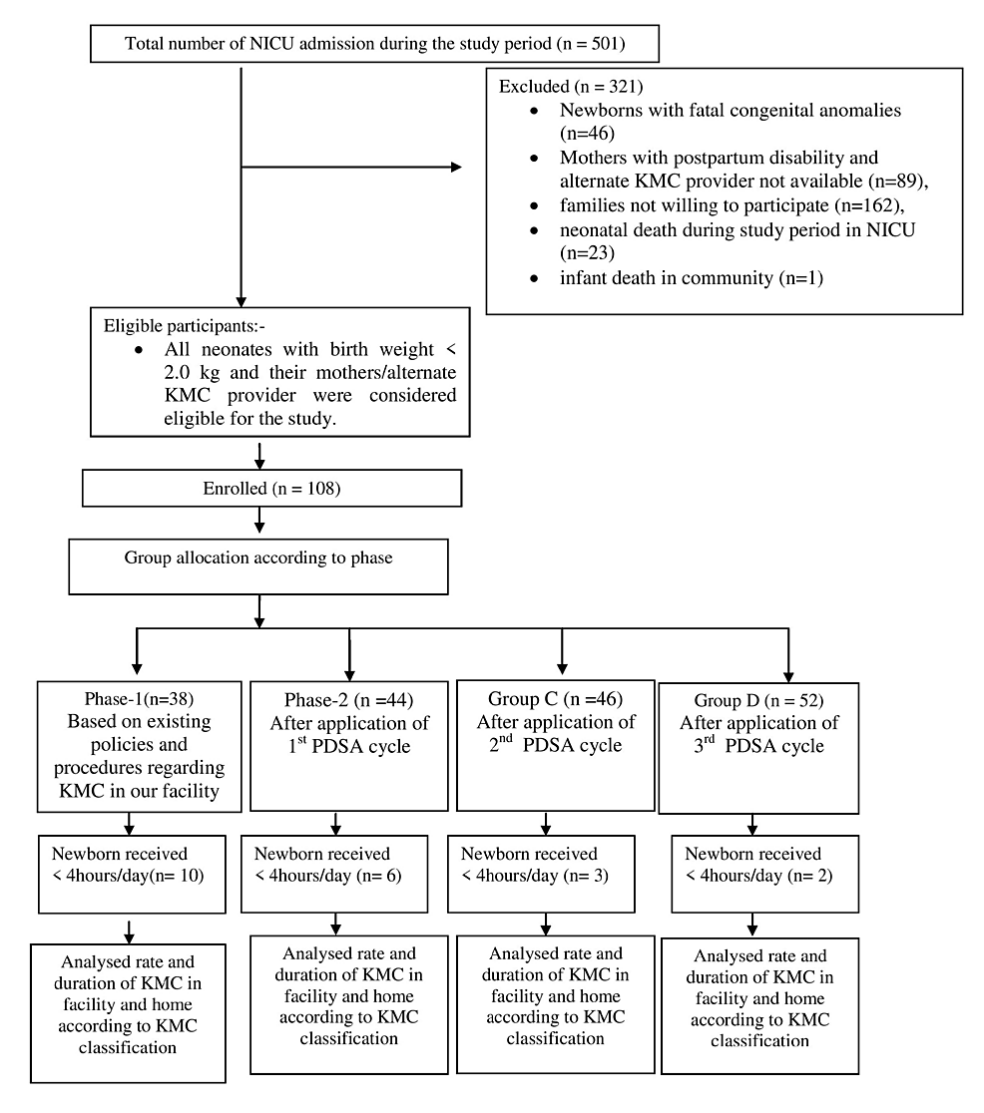
Participant Flow Diagram NICU: Neonatal intensive care unit; PDSA: plan-do-study-act; KMC: kangaroo mother care

A total of 501 newborns were admitted during the study period. Figure 2 depicts the flow diagram of participant recruitment in this study. After exclusions, 180 neonates with birth weight < 2.0 kg and their mothers/alternate KMC providers were considered eligible for the study. Out Of these, 180 maternal/alternative KMC providers-infant dyads 38, 44, 46, and 52-enrolled at stages 1, 2, 3, and 4, respectively. But 21 neonates received KMC < 4 hours/day. The participants’ birth weight and "the weight at KMC start" were 1.36±0.30 and 1.32±0.26 kg, respectively. The mean age of the mother and father was 24.62±3.93 and 27.58±4.10 years, respectively. In most infants, KMC was initiated within four days of life. The baseline characteristics of enrolled newborns are summarized in Table 2. 

**Table 2:** Baseline Characteristics of Study Participants #Data presented as Mean ± SD or Number (Percentage) KMC: Kangaroo mother care

Weight	At Birth (in kilograms)	1.36±0.30
	Weight at KMC start (in kilograms)	1.32±0.26
Baby Gender	Male	96
	Female	84
Age (Years)	Father	27.58±4.10
	Mother	24.62±3.93
Religion	Hindu	144
	Muslim	36
Initiation of KMC (age in days)	Day-1	33 (18.33)
	Day-2	33 (18.33)
	Day-3	39 (21.67)
	Day-4	36 (20.00)
	Day-5	24 (13.33)
	Day-6	09 (5.00)
	Day-7	06 (3.33)

**Figure 3:**
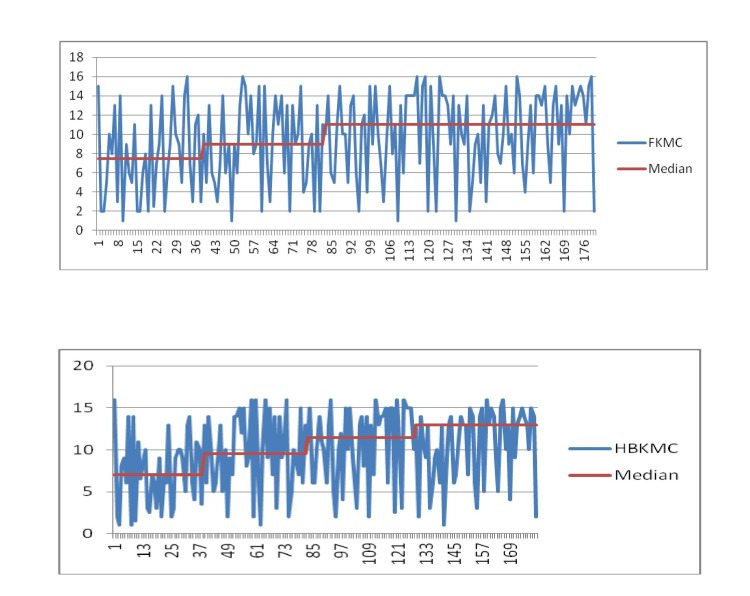
Run Chart Showing Kangaroo Mother Care at Facility (FKMC) and Home-Based Kangaroo Mother Care (HBKMC)

According to KMC classification, 31% have continuous KMC in the institution, followed by 24% long KMC, 26% extended KMC, and 18% short KMC. After three PDSA cycles, HBKMC was 38.88% continuous KMC, followed by 24.22% long KMC, 20.55% extended KMC, and 16.11% short KMC. Continuous KMC was improved from 21% to 46% at the institute and 16% to 50% at home from phase 1 to phase 4 of study after implementation of three sets of interventions in three PDSA cycles. The phase-by-phase KMC rate and duration were improved after the application of the PDSA cycles, and this was maintained in HBKMC as well, but it was statistically not significant (Figure 3, Table 3). 

**Table 3:** KMC Duration and Rate Before and After the PDSA Cycle at the Institute and at Home #Data presented as number (percentage) KMC: Kangaroo mother care; PDSA: plan-do-study-act

KMC Classification	Phase 1		Phase 2		Phase 3		Phase 4		p value	Total		p value
	At Institute	At Home	At Institute	At Home	At Institute	At Home	At Institute	At Home		At Institute	At Home	
Continuous	8 (21.05)	6 (15.78 )	14 (31.82)	16 (36.36)	20 (43.48)	22 (47.83)	24 (46.16)	26 (50.00)	0.39	56 (31.11)	70 (38.89)	>0.05
Long	9 (23.69)	10 (26.32)	12 (27.21)	12 (27.28)	12 (26.09)	10 (21.74)	14 (26.92)	12 (23.08)	0.39	44 (24.44)	44 (24.44)	
Extended	10 (26.32)	10 (26.32)	10 (22.75)	11 (25.00)	08 (17.39)	08 (17.39)	08 (15.38)	08 (15.38)	0.39	47 (26.11)	37 (20.56)	
Short	11 (28.94)	12 (31.58)	08 (18.22)	05 (11.36)	06 (13.04)	06 (13.04)	06 (11.54)	06 (11.54)	0.39	33 (18.34)	29 (16.11)	
Total	38 (100)	38 (100)	44 (100)	44 (100)	46 (100)	46 (100)	52 (100)	52 (100)	NA	180 (100)	180 (100)	

The duration of KMC was improved statistically significant in the group where both the mother and alternative KMC provider were giving KMC to LBWIs as compared to other groups. The duration of KMC in the urban communities was statistically significant. The educational level of the father or mother did not carry any statistical significance (Table 4). The duration of KMC was statistically significant in the group where parents were consulted by doctors. The duration of HBKMC was statistically significant in the group where the parents were assisted by a CHW (Table 4). The “episodes of illness requiring hospitalization” had an adverse effect on the duration of KMC and were also statistically significant. However, only 11.67% of LBWIs had episodes of illness who received KMC ≥4 hours in the institution as well as at home. The proportion of breastfed LBWIs was significantly higher than that of non-breastfed LBWIs, and most babies were directly breastfed. The duration of KMC and weight gain positively correlated and was statistically significant. 

**Table 4:** Comparison of Other Outcomes in the Study Groups *p<0.05 KMC: Kangaroo mother care; CHW: child health care worker

Outcome			Number with Percentage	KMC in Hours Duration (Mean±SD)	p value
Breast Feeding	Yes		141 (78.33%)	-	-
	No		39 (21.67%)	-	
Method of Feeding	Breast Feeding		142(88.33%)	-	-
	Cup Feeding		38(11.67%)	-	
Urban/Rural	Urban		54(30%)	11.86±3.20	<0.0001*
	Rural		126(70%)	08.63±2.16	
KMC Counseling	Nursing Officer		75 (41.67%)	8.54±2.30	<0.0001*
	Doctors		105 (58.33%)	9.91±3.20	
CHW Assistance	Yes		75(41.46%)	10.80±2.76	<0.0001*
	No		105(58.34%)	08.50±2.57	
KMC Provider	Mothers		12(6.67%)	7.0±5.62	<0.0001*
	Others		12(6.67%)	9.5±1.03	
	Mother + Others		135(75.00%)	9.77±2.51	
Education (Mother)	Illiterate		48 (26.67%)	9.57± 3.08	>0.05
	Middle school		24 (13.33%)	7.33± 0.97	
	Secondary School		66 (36.67%)	8.70 ±3.39	
	Senior Secondary School		12 (06.67%)	8.40± 2.41	
	Graduate		24 (13.33%)	8.00 ±1.19	
	Post Graduate		06 (03.33%)	11.00± 5.48	
Education (Father)	Illiterate		27 (15.00%)	8.33 ±3.01	>0.05
	Middle school		15 (08.33%)	8.00± 0.00	
	Secondary School		48 (26.67%)	9.38 ±3.17	
	Senior Secondary School		24 (35.00%)	9.50 ±3.02	
	Graduate		63 (35.00%)	7.78± 2.60	
	Post-Graduate		03 (35.00%)	16.00± 0.00	
KMC Duration	Facility-initiated KMC		159(88.33%)	9.54±2.88	0.975
	Home-based KMC		159(88.33%)	9.55±2.89	
Sickness Episode	Yes	KMC 0-4 hours	21(11.67%)	6.11±2.69	<0.0001*
		KMC≥4hours	18 (10.00%)		
	No		141(78.33%)	9.98±2.63	

## Discussion

A quality improvement study to improve home-based KMC was conducted by using three sets of interventions through PDSA cycles in four phases. This study was conducted in JK Lon Hospital, Jaipur, Rajasthan, India, and nearby districts. Initially, all four phases were implanted in JK Lon Hospital, Jaipur as FKMC. Later, the same phases were implemented in the community of the nearby district as HBKMC. Continuous KMCs were improved from 21% to 46% at the facility and 16% to 50% at home from phase 1 to phase 4 of the study after the implementation of three sets of interventions in three PDSA cycles. The phase-by-phase KMC rate and duration were improved after the application of the PDSA cycles, and this was maintained in HBKMC, although it was statistically not significant. The duration of IKMC (median) was 7.5 hours/day in the per-implementation phase (phase 1) which was increased to 9.0, 11.0 h/day in phases 2 and 3 after the implementation of one and two PDSA cycles. Although the implementation of PDSA cycle 3 did not improve the duration of IKMC from stage 3 to stage 4, it remained the same i.e. 11 hours/day (median).

The duration of HBKMC (median) was 7 h/day in the per-implementation phase (phase 1) which was increased to 9.5, 11.5, and 13 hours in phases 2, 3, and 4 after the implementation of three PDSA cycles. Similar findings to our study were also noted in previously published quality improvement studies but these studies were conducted to improve the duration of KMC at the institution [14,15,16]. In the study by Joshi et al., using three PDSA cycles increased the duration of KMC (median) from 3.2 hours per day to 7.5 hours per day at the institution [14]. In the study by Ramachandrappa et al., using three PDSA cycles, the duration of KMC at the institute was increased from 2.7 hours/baby/day to 7.8 hours/baby/day [15]. In the study by Jegannathan et al., the duration of KMC was improved from 4.6 hours/baby/day to 16.6 hours/baby/day by implementing seven PDSA cycles [13].

An observational study using in-depth interviews, key informant interviews, and group discussions from communities in east-central Uganda was conducted to examine barriers and facilitators to the continuation of KMC at home. This study found that despite the widespread acceptance of KMC by community health workers, mothers practiced KMC for less time at home than in the hospital [17]. In contrast, in our study, the KMC duration was maintained in HBKMC. The mean duration of KMC at home in our study was 9.55±2.89 h which was comparable with facility-initiated KMC which was 9.54±2.88 h. In contrast, in another study, HBKMC varied from 3-8 h in India where antenatal and postnatal counseling was provided by HCWs in the community [18]. In our study, infants who received KMC in NICU had 78% exclusive breastfeeding at the time of hospital discharge. Similar findings to our study were also noted in previously published studies [19,20,21,22]. In our study, the duration of KMC was statistically more significant when the doctor provided counseling to the KMC provider. Similar findings to our study were also noted in the previously published study [23]. In our study, the duration of KMC was statistically more significant in the presence of CHW assistance. Similar findings to our study were also noted in previously published studies [18].

## Conclusions

Sets of intervention packages based on needs analysis using the PDSA cycle were able to improve the rate and duration of KMC in the hospital and at home. CHWs can play an important role in the duration of HBKM. Continuous communication with the KMC provider also improves the HBKMC duration. The duration of KMC can be improved by counseling, policymaking, and supporting the KMC provider based on needs analysis in the institution and community. CHW assistance can play an important role in maintaining continuous KMC in the community.

## Appendices

WHO-World Health Organization

UNICEF- United Nations International Children's Emergency Fund

KMC- Kangaroo Mother Care

IKMC-Immediate Kangaroo Mother Care

HBKMC-Home-Based Kangaroo Mother Care

FKMC- Facility-initiated KMC

CMC-Conventional Mode of Care

NICU-Neonatal Intensive Care Unit

PDSA- Plan-Do-Study-Act

LBW-Low Birth Weight

LBWI-Low-Birth-Weight Infant

CPAP-Continuous Positive Airway Pressure

IDI- In-Depth Interview

KI- Key Informant Interview

CHW- Community Health Worker
